# The Shape of Relations to Come: Multidimensional Analyses of Complex Human Behavior

**DOI:** 10.1007/s40732-023-00575-9

**Published:** 2024-01-09

**Authors:** Lee Mason, Alonzo Andrews, Maria Otero, Kimberly James-Kelly

**Affiliations:** 1 Child Study Center, Cook Children’s Health Care System, 1300 West Lancaster Avenue, Fort Worth, TX 76110, USA; 2 Burnett School of Medicine, Texas Christian University, Fort Worth, TX, USA; 3 Professional and Continuing Education, University of Texas at San Antonio, San Antonio, TX, USA; 4 Department of Behavior Analysis, University of North Texas, Denton, TX, USA; 5 Jane Justin School, Cook Children’s Health Care System, Fort Worth, USA

**Keywords:** Radar chart, Multidimensional analysis, Polygonal profiles, Shape descriptors

## Abstract

Science, understood to be the behavior of scientists, falls within the purview of behavior analysis. All scientists use scientific instruments to study a natural phenomenon, and for the behavior analyst, perhaps no tool is more important than the graph used to show changes in level, trend, and variability, and upon which behavior analysts make data-based decisions. Modern behaviorism as we know it dates back to the development of the cumulative recorder first developed in the 1930s. Though revolutionary to the science of behavior, two-dimensional graphs may be limited in application for analyzing complex human behavior. In the current article, we conceptualize verbal behavior as a multidimensional field of environmental relations, and introduce the use of multi-axial radar charts for its visual and quantitative analysis. From there, we survey the use of radar charts toward advancing a behavior-analytic understanding of human language and cognition. We demonstrate the use of radar charts for calculating simple shape descriptors as a quantitative measure of dynamic interactants, and show how they can be used to measure change over time.

An analysis of the relationship between two quantities is a cornerstone of any scientific investigation, whether it be a comparison of naturally occurring properties (e.g., stress-strain) or the functional relationship between variables (e.g., force-displacement). At the most basic level, scientists measure the extent to which one quantity is affected by a change in the other. As the subject matter becomes more complex, however, our analyses must concomitantly adapt.

The cumulative recorder was a revolutionary development toward a natural science of behavior, because it made amenable both visual and quantitative analysis of environmental relations (Morris & Smith, 2004). Cumulative records provided the first real-time analysis of environmental relations, and were instrumental to the discovery of schedules of reinforcement (Lattal, 2004). Although the shape and display of the lines have changed over the last century, a perusal of the experimental or applied literature on behavior analysis reveals that time-series line graphs—those on which a dimension of behavior is plotted on one axis, and a dimension of time is plotted on another—serve as the primary means of analysis.

The study of language within the natural science of behavior analysis serves as a prime example. Researchers examining verbal behavior have generally approached functionally distinct verbal operants as categorical variables. According to Boolean logic, a given verbal response is classified as mand, tact, echoic, or intraverbal, with each separate operant giving way to a mutually exclusive line of research. As Michael et al. (2011) noted, “… a common preoccupation of students is to try to classify utterances as one or another verbal operant on the assumption that the example must be exclusively one type” (p. 4). Although this approach bears some utility for a basic analysis of verbal behavior, it fails to capture the complexity and dynamics of human language.

The segregation of verbal operants fits nicely into the standard analytic framework, with the frequency of responses on the ordinate and a sequence of events on the abscissa, but such a molecular approach to studying language obfuscates the history of reinforcement that supports more complex interactions. Although simple molecular analyses are useful for examining simple responses in highly controlled settings, a study of complex behavior must also account for supplementary sources of control—both present and historical—that work to support or compete with the prevailing contingencies. For example, the nature of researching emergent language precludes an analysis of the speaker’s response rate. Instead, researchers often resort to pre/posttesting to demonstrate this phenomenon. Figure 1, redrawn from the published literature on language emergence, provides a representative exemplar of this approach.

The vertical dashed line represents not only a phase change, but an undocumented history of conditioning that ostensibly accounts for the change in behavior. Throughout the literature, this unknown variable has been tact training (e.g., Miguel & Kobari-Wright, 2013), listener training (e.g., Petursdottir et al., 2008), mand training, (e.g., Egan & Barnes-Holmes, 2009), or intraverbal training (e.g., Ingvarsson et al., 2012). It should be noted that the emergence of untrained relations is inconsistent across studies (see Grow & Kodak, 2010; Wooderson et al., 2022). For the studies in which untrained relations successfully emerge, two-dimensional graphs do little to clarify the participant’s contingency history. In what ways has the participant’s relational network transformed over the course of the phase change? It is clear that something is missing in the analysis of language acquisition.

Sidman (1979/2010) noted “… that an analysis of stimulus control always involves an inference.… Unlike individual stimuli and responses, controlling relations are not directly observable” (p. 133). This certainly appears to be the case with research on emergent verbal behavior. Although the narratives that accompany each investigation provide additional details on their respective methodology, visual analysis of the data—the hallmark of single-case experimental research—breeds a large amount of inference between pretest and posttest outcomes. Though a sufficient literature base exists to demonstrate the emergence of untrained verbal relations, there is still far too much inference to demonstrate a functional relationship with its environmental determinants.

Grow and Kodak (2010) called for additional research on the skills that are assessed and targeted in early intervention programs to maximize the acquisition of emergent verbal behavior. There can be no doubt that the individual differences of research participants play an important role in their ability to derive stimulus relations, but a comprehensive picture of interdependent variables presupposes scientific explanation. Before researchers continue looking for a functional relationship between the acquisition of one verbal operant and the emergence of others, we should note that using a two-dimensional line graph to do so may be as effective as trying to clear away the darkness by thrusting it aside with one’s hands (Watts, 1951). “Perhaps more importantly,” observed Michael et al. (2011) “if one fails to consider multiple control, one’s interpretations of verbal behavior are likely to be conspicuously inadequate” (p. 4).

## Continuum of Control

Natural biological materials exhibit a multitude of mechanical and functional properties. For example, spider silk is extraordinarily strong, yet elastic; mollusk shells are light though tough; and bird feathers and porcupine quills are both rigid and durable (Meyers et al., 2013). Stress-strain curves are frequently used to describe the relationship between two such properties (Ashby, 2000), but what if investigators are interested in examining combinations of properties or more complex interactions?

Researchers studying other complex systems have encountered similar difficulties when attempting to examine molecular relationships. Wilson and Wilson (2007) describe a multilevel theory of evolutionary selection. Porter and Niksiar (2018) introduce a multidimensional analysis of biological materials. Although behavior analysis has a rich history of research on multiple schedules of reinforcement (Ferster & Skinner, 1957), only more recently have researchers begun to examine complex behavior. An analysis of complex verbal behavior similarly benefits from treatment as a continuous variable. Rather than being mand or tact, echoic or intraverbal, a given verbal response is often simultaneously a member of two or more sets. Multiple control is the rule rather than the exception (Michael et al., 2011).

A radical departure from Boolean logic (i.e., dichotomous values of 0 or 1), the advent of fuzzy logic incorporates the range of variables existing between real numbers (i.e., continuous values from 0 to 1). Fuzzy logic changed the nature of computational electronics, contributing to the development of “smart” technologies (e.g., phones and kitchen appliances), automotive systems (e.g., antilock brakes and traction control), air conditioning, and artificial intelligence (Rushdi et al., 2015). No longer mutually exclusive 0s and 1s, fuzzy values are considered in terms of degrees.

**Fig. 2:**
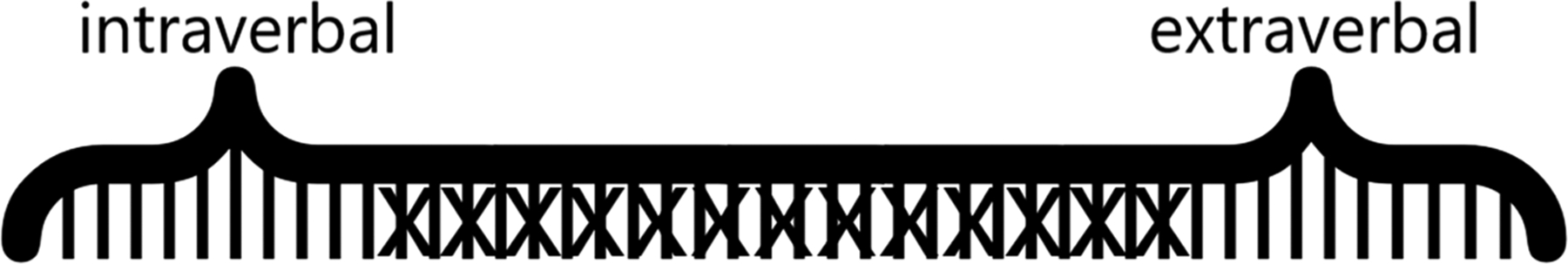
A Representation of Vargas’s (1982) Intraverbal-Extraverbal Continuum of Control over Verbal Behavior

Akin to complex numbers, which exist on a coordinate plane constructed of real and imaginary number lines, complex verbal behavior can be expressed on a coordinate plane comprised of intraverbal and extraverbal sources of control. Although intraverbal behavior is that which is under the control of other verbal behavior, extraverbal behavior is, by definition, under the control of nonverbal events. Vargas (1982) described a *continuum of control* that extends from intraverbal relations at one end, to extraverbal relations at the other (see Fig. 2).

Vargas’s notion of a *continuum of control* points to the mutual entanglement of the verbal operants. The control over a given verbal response may be more or less intraverbal while concomitantly less or more extraverbal, but rarely—if ever—mutually exclusive of the other (Belisle et al., 2021; Fryling, 2017).

**Fig. 3:**
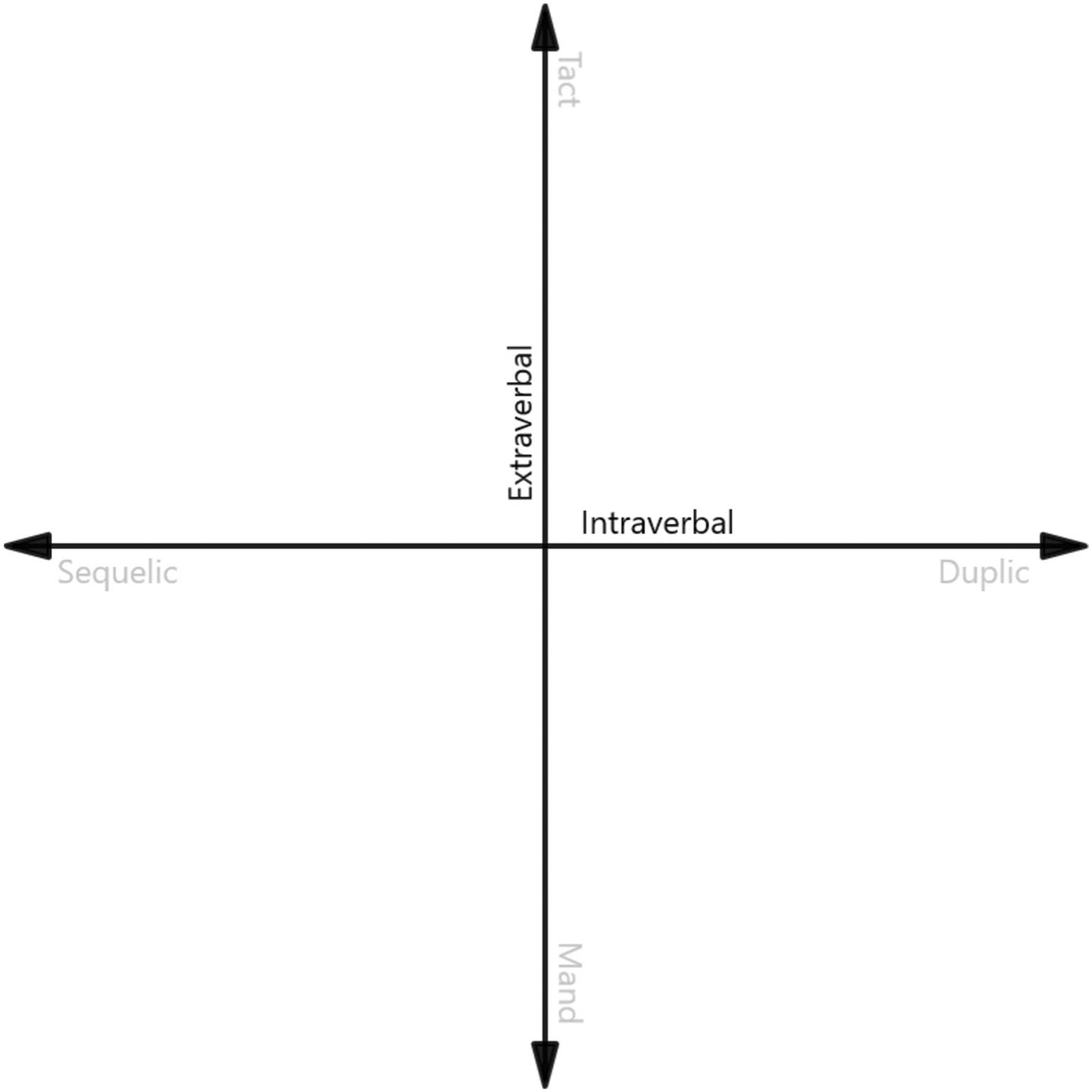
The Intersection of Intraverbal and Extraverbal Continua of Control Yields a Complex Plane for Analyzing Complex Verbal Behavior

Though Vargas (1982) described a single continuum between intraverbal and extraverbal sources, we argue that—for analyzing complex verbal behavior—the relationship is better expressed as the intersection of intraverbal and extraverbal control. Intraverbal control ranges from the presence of point-to-point correspondence (i.e., duplic) to the absence of point-to-point correspondence (i.e., sequelic). Extraverbal control ranges from the presence of a nonverbal stimulus (i.e., tact) to the absence of a nonverbal stimulus (i.e., mand).^1^ These two continua of controlling variables converge perpendicularly to create a Cartesian coordinate system for analyzing complex verbal behavior (see Fig. 3).

**Fig. 4:**
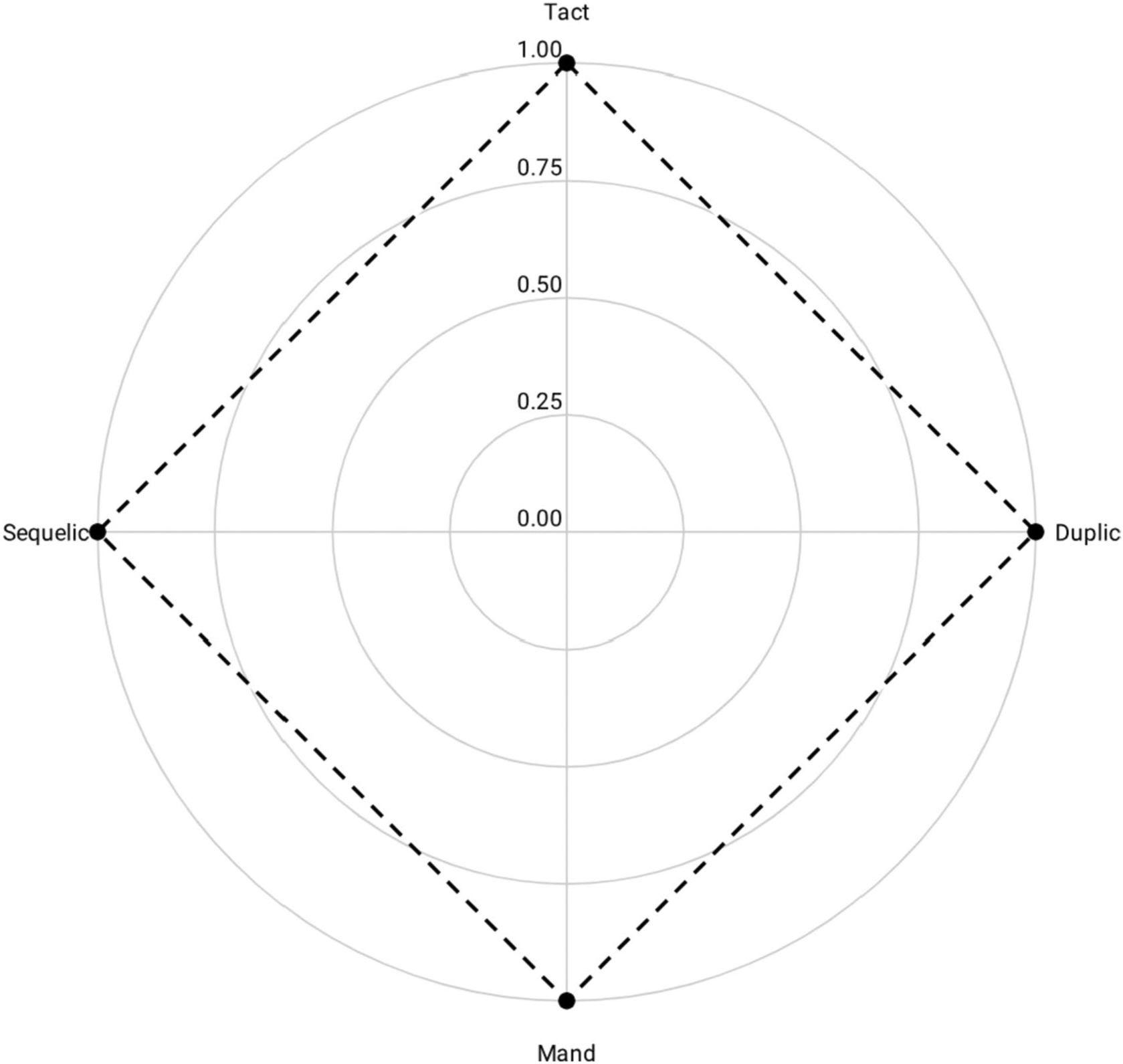
Connecting Adjacent Data Points Creates a Closed Polygonal Language Profile for Visual and Quantitative Analysis

The resulting radar chart affords an analysis of epicon-textual relations, those both present (i.e., contemporary or explicit) and absent (i.e., historic or derived). For example, the frequency data from a verbal operant experimental (VOX) analysis, in which responses are induced across strictly controlled environmental relations (see Mason & Andrews, 2019), can be plotted on each of the corresponding radial axes. Drawing a straight line between each adjacent point on the radar chart then creates a unique polygonal language profile that is amenable to analysis using a compilation of shape descriptors (Peura & Iivarinen, 1997; see Fig. 4).

Unlike bar charts or pie charts, which emphasize discrete categorical variables, radar charts emphasize the continuity between extraverbal and intraverbal sources of control; depicting how a change in one parameter affects the entirety of the polygonal profile. Radar charts are sensitive to the multidimensionality of complex systems that account for combinations of input and output variables. As Michael et al. (2011) explained, “In convergent multiple control, more than one variable strengthens a response of a single topography, whereas in divergent multiple control, one variable strengthens more than one response” (pp. 5–6). As a result, a complete analysis of complex verbal behavior necessitates a framework that allows for exploring continuous variables across a continuum of control.

What follows is a survey of applications of radar charts to the analysis of human language and cognition. We begin by examining explicit verbal relations and progress through simple relational frames to higher-level interactants. For each scenario, different assessments methodologies can be found within the relevant literature. Across scenarios, we plot the frequency of discriminated responses on the corresponding radial axis of the radar chart to create distinct polygonal profiles for quantitative and visual analysis.

## Shape Descriptors of Stimulus Relations

The tension between the ends of each continua creates the framework for conducting a multidimensional analysis on a Cartesian coordinate plane. Porter and Niksiar (2018) recommended the use of multi-axial radar charts for the performance mapping of natural biological systems. In particular, radar charts are useful for complex analyses consisting of a concurrent analysis of multiple mechanical or functional properties. Extending the work of Porter and Niksiar (2018), we employ the radar chart for visually analyzing the gestalt verbal repertoire, and perform quantitative analyses of the relevant shape descriptors for each resulting polygonal profile. A multitude of shape descriptors are commonly employed for 2D and 3D image analysis and pattern recognition, but for our purposes we will limit the discussion to three moment-based attributes: area, centroidal distance, and first moment of area.

For each of the following polygonal profiles, we began by calculating the area (*A*) of the polygonal profile using the following formula:

$$A=\frac{1}{2}\left(\frac{\left|x_{1}\right|}{\left|y_{1}\right|}\frac{\left|x_{2}\right|}{\left|y_{2}\right|}+\frac{\left|x_{2}\right|}{\left|y_{2}\right|}\frac{\left|x_{3}\right|}{\left|y_{3}\right|}+\ldots +\frac{\left|x_{n}\right|}{\left|y_{n}\right|}\frac{\left|x_{1}\right|}{\left|y_{1}\right|}\right)$$

where the vertices are expressed in terms of the absolute value of Cartesian coordinates (*x, y*).

The geometric center of a mass, known as the centroid, is the arithmetic mean position of all the points in the figure. We used the following formulas to calculate the polygonal profile’s Euclidean distance from the horizontal axis:

$$\bar{x}=\frac{\sum A_{i}x_{i}}{\sum A_{i}}$$

and vertical axis:

$$\bar{y}=\frac{\sum A_{i}y_{i}}{\sum A_{i}}$$

respectively. This allowed us to pinpoint the centroid of the polygonal profile on the radar chart, along with the centroidal distance (*R*) from the origin of the coordinate system:

$$R=\sqrt{{\bar{x}}^{2}+{\bar{y}}^{2}}$$

The centroid and centroidal distance allow us to begin to understand the skewedness of the verbal repertoire, giving us an understanding of prepotent sources of control. With all the prerequisites in place, the magnitude of the first moment of area (*Q*) is calculated in terms of the profile area (*A*) and the distance from the origin to the centroid (*R*). The multidimensional performance is defined as the profile’s normalized first moment of area relative to the circumference of the property space (*C*)^2^:

Q=A(C−R)

This metric is analogous to a distribution function within statistics or the measure of inertia within physics (Flusser et al., 2009). The continuity between other natural sciences and behavior analysis (see Belisle & Dixon, 2020; Donahoe, 2021; Timberlake, 1999) affords the extension of this technology toward examining complex human behavior such as language and cognition.

First moment of area is a unique metric in that it summarizes both the size and distribution of the polygonal profile. A polygonal profile may have a large *A* (i.e., many different responses) along with a large *R* (i.e., the distribution is significantly skewed; see Mason et al., 2022). This combination would yield a small value for *Q*. Likewise, it is possible for a polygonal profile to have a small *A* (i.e., few responses) in conjunction with a small *R* (i.e., the distribution is relatively proportional). This combination would also result in a small value for *Q*.

First moment of area increases in value as the area of the polygonal profile increases (i.e., large *A*), whereas the centroidal distance decreases (i.e., small *R*). That is, fluent speakers tend to show a broad variety of responses that are equally balanced across the different verbal operants when sampled in isolation. A large *Q* value is foundational to the relational flexibility required of dynamic environmental interactions (Kelly, 2020; O’Toole & Barnes-Holmes, 2009).

## Measuring Verbal Relations

**Fig. 5:**
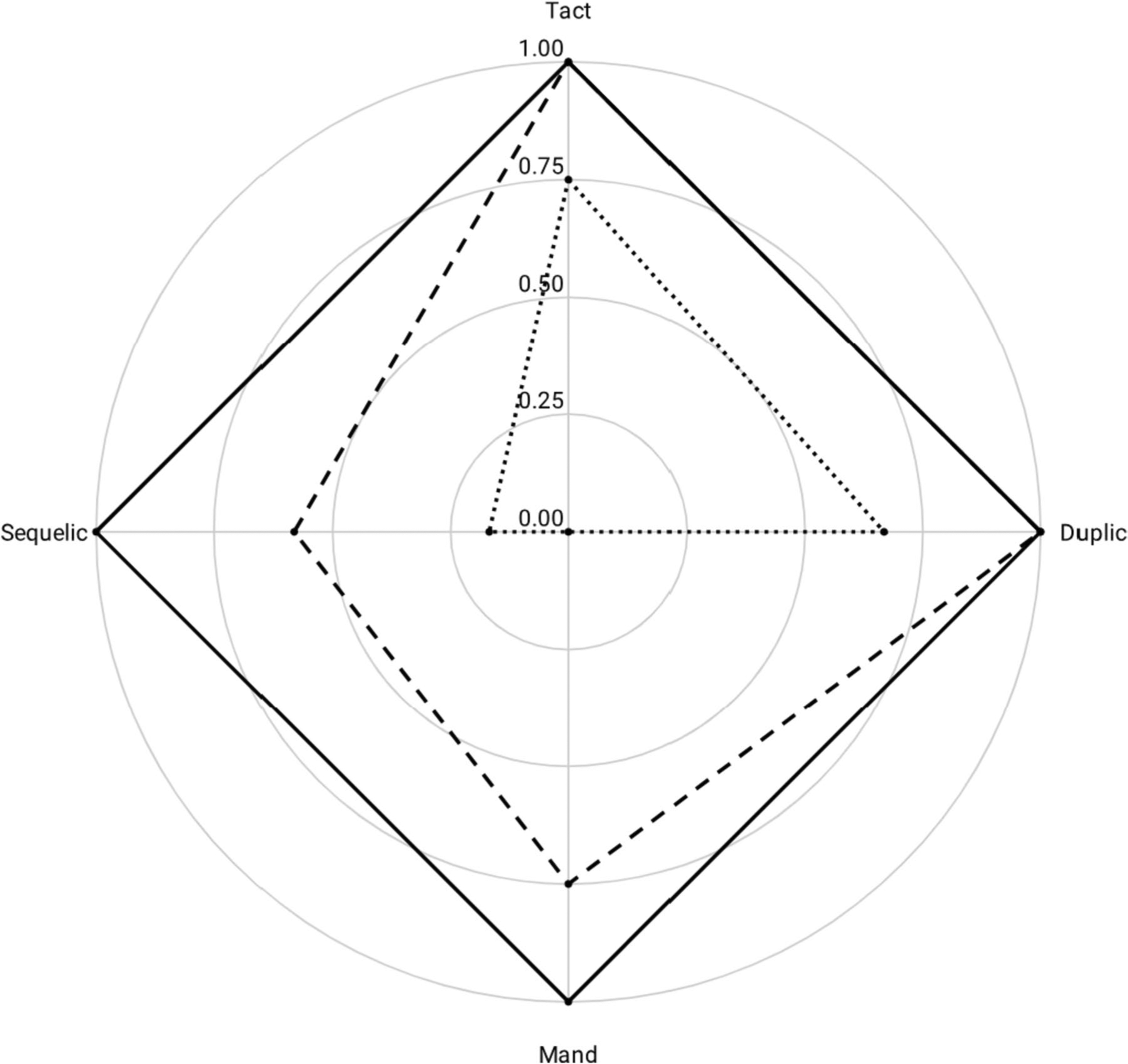
A Multi-Axial Radar Chart Resulting from the Intersection of Intraverbal and Extraverbal Continua of Control Showing the Results of Two VOX Analyses for a Boy with Autism over 6 Months of Early Intensive Behavioral Intervention. *Note*: Initial assessment, dotted line; Reassessment – dashed line; Terminal model – solid line

Multidimensional radar charts are particularly useful for measuring change over time. Plotting frequency data on a radar chart emphasizes the dynamic nature of environmental control, and allow researchers and practitioners to observe the extent to which stimulus relations across the repertoire covary with the behavior of interest. An example of this can be seen in Fig. 5, on which we have plotted a functional language sample of a 4½-year-old boy with autism who received early intensive behavioral intervention (EIBI) over the course of 6 months. The results of both the initial assessment and 6-month reassessment showed skewed response distributions, though the speaker’s verbal repertoire displayed greater proportionality over time.

The dotted line shows the results of initial language assessment. Using the formulas above, we found *A* = 0.31, *R* = .30, with centroid located at $\bar{x}=.17$, $\bar{y}=.25$. This allowed us to calculate the first moment of area for the initial language profile at *Q* = 0.22. The dashed line shows the results of a reassessment conducted after 6 months of EIBI: *A* = 1.39, *R* = .16 (.14, .08), *Q* = 1.16. Between the two assessments, first moment of area increased by 0.94.

The solid line shows the maximum possible value of a fluent speaker. The relative balance of the The solid line shows the maximum possible value of a fluent speaker. The relative balance of the verbal repertoire places the centroid at $\bar{x}=0$, $\bar{y}=0$ with *R* = 0, and *A* = 2. We can then calculate the maximum *Q* = 2. The proportional strength across all four verbal operants is indicative of neurotypical language development for speakers over 3 years of age, which allows *Q* = 2 to serve as a benchmark for measuring the language of speakers with autism and other verbal behavior disorders (see Table 1).

## Measuring Derived Stimulus Relations

**Fig. 6:**
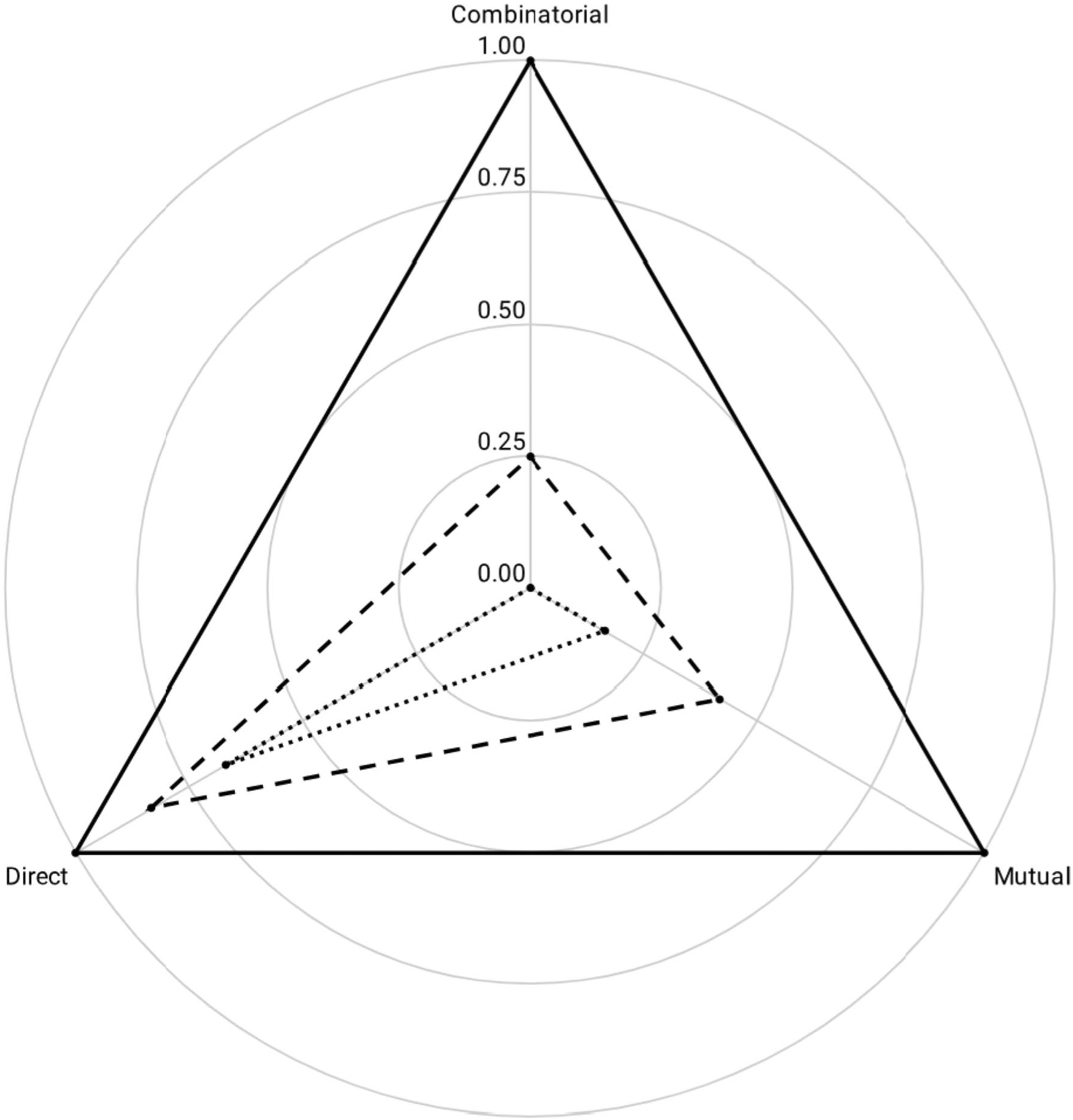
A Multi-Axial Radar Chart Displays Relational Frames of Coordination on which We have Plotted the Results of Relational Operant Analyses of a Six-Year-Old Boy with Autism before (dashed line) and after (solid line) One Year of Early Intensive Behavioral Intervention*. Note*: Initial assessment, dotted line; Reassessment—dashed line; Terminal model—solid line

As a speaker’s language skills become more complex, the axes of the radar chart may be modified to account for derived stimulus relations. A similar analytic approach is applicable to the more abstract measures of human cognition described by relational frame theory (RFT; Hayes et al., 2001; Barnes-Holmes et al., 2020). Here, we develop a polygonal relational profile based on the frequency of responses to a series of logical syllogisms counterbalanced across frames of coordination.^3^ Figure 6 displays a multi-axial radar chart comparing the frequency of directly trained, mutually entailed, and combinatorially entailed responses of a child with autism before and after 12-mo of EIBI (Cassidy et al., 2016; Kirsten & Stewart, 2022).

Visual analysis shows a significant difference in the speaker’s arbitrarily applicable relational responding across time. As above, shape descriptors can be found for each polygonal profile. Prior to intervention (dotted line), the speaker’s relational profile largely consisted of direct relations, with few mutual and no combinatorial relations. The relational profile measured *A* = 0.05, with *R* = 0.27, which yielded *Q* = 0.03. After 1 year of intervention (dashed line), the speaker’s relational profile is larger and shows greater proportionality. The updated relational profile measured *A* = 0.29, and *R* = 0.17, which equates to *Q* = 0.23. The difference between these two relating repertoires was calculated as *Q* = 0.20. Note that this same technique can be used to compare a speaker’s relational profile against a perfectly balanced model (solid line), in which *Q* = 1.30. In addition, the frame of coordination can be substituted for other families of relational frames (e.g., distinction, containment, and temporality; see Table 2).

## Measuring Deictic Relations

**Fig. 7:**
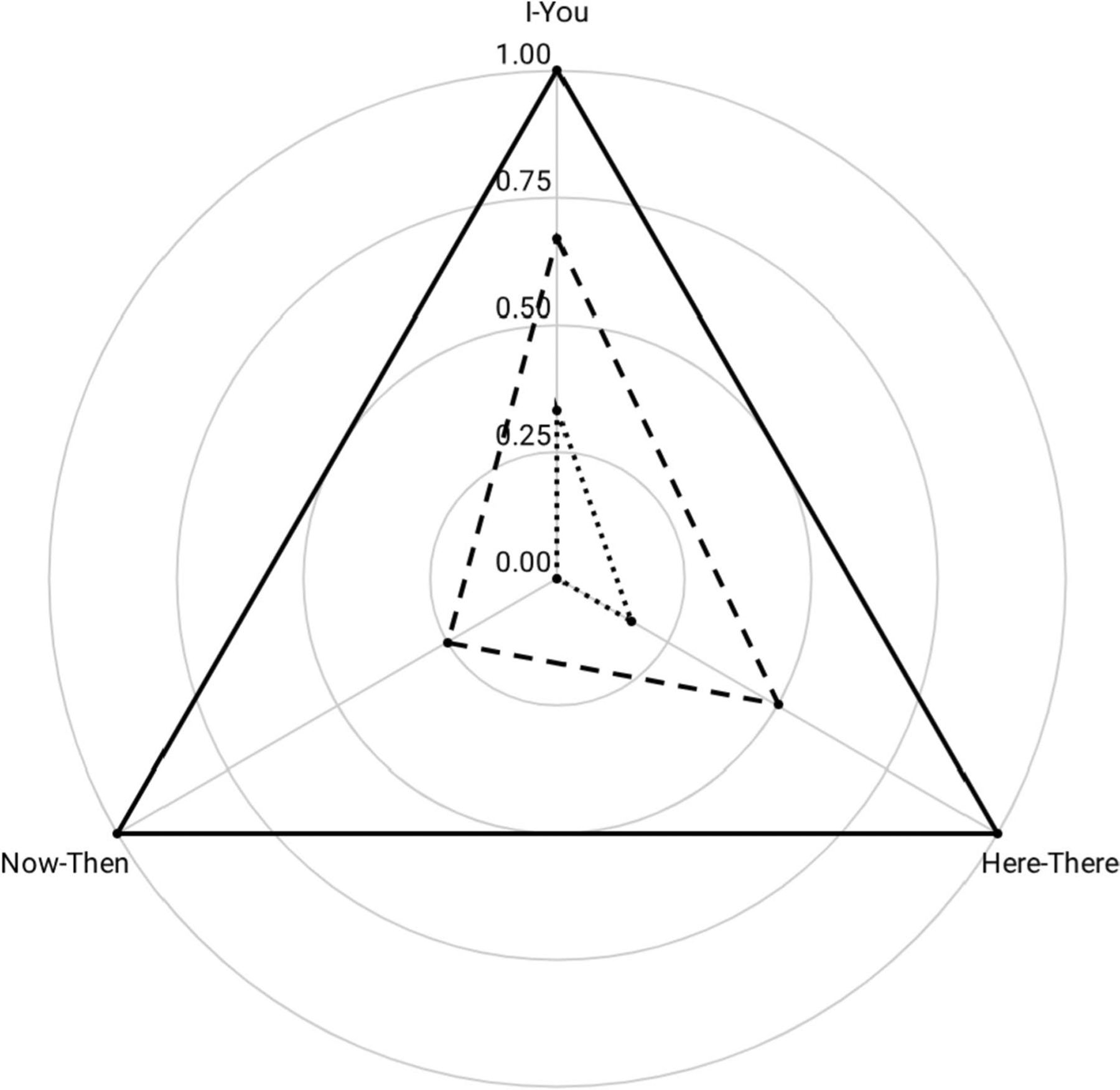
A Radar Chart Displaying Proportionate Levels of Strength among the Three Core Deictic Relations of the Self. *Note*: Initial assessment, dotted line; Reassessment – dashed line; Terminal model – solid line

Just as verbal behavior allows the speaker to extend their control of the environment across time and space, Harte and Barnes-Holmes (2021) identified three core deictic relations involved in locating oneself in time and space: the interpersonal relation (i.e., I–You), the spatial relation (i.e., Here–There), and the temporal relation (i.e., Now–Then). Deictic frames are considered a more advanced form of relational responding that involves both relating relations and relating entire relational networks (Kavanagh et al., 2019). A multidimensional analysis of the deictic relation provides a precise measure of the extent to which an individual relates oneself to others within a particular spatial–temporal context. Figure 7 shows a representative example of deictic relations developing over time, against a model showing proportional levels of interpersonal, spatial, and temporal relations.

The frequency of discriminated relations of the self can be plotted on each axis, allowing for the comparison of growth over time. The initial assessment (dotted line) shows *A* = 0.02, *R* = .10 (.05, .08), for *Q* = 0.02. A reassessment (dashed line) shows *A* = 0.27, *R* = .12 (.07, .10), for *Q* = 0.24. The deictic repertoire grew by *Q* = 0.22.

On a normalized scale, the maximum deictic profile (solid line) has an area of 1.30, centroidal distance of 0, and first moment of area of 1.30. Plotting the observed data against this model of the self may help to identify areas in need of clinical intervention (see Table 3).

## Measuring Hyperdimensional Multilevel Frameworks

**Fig. 8:**
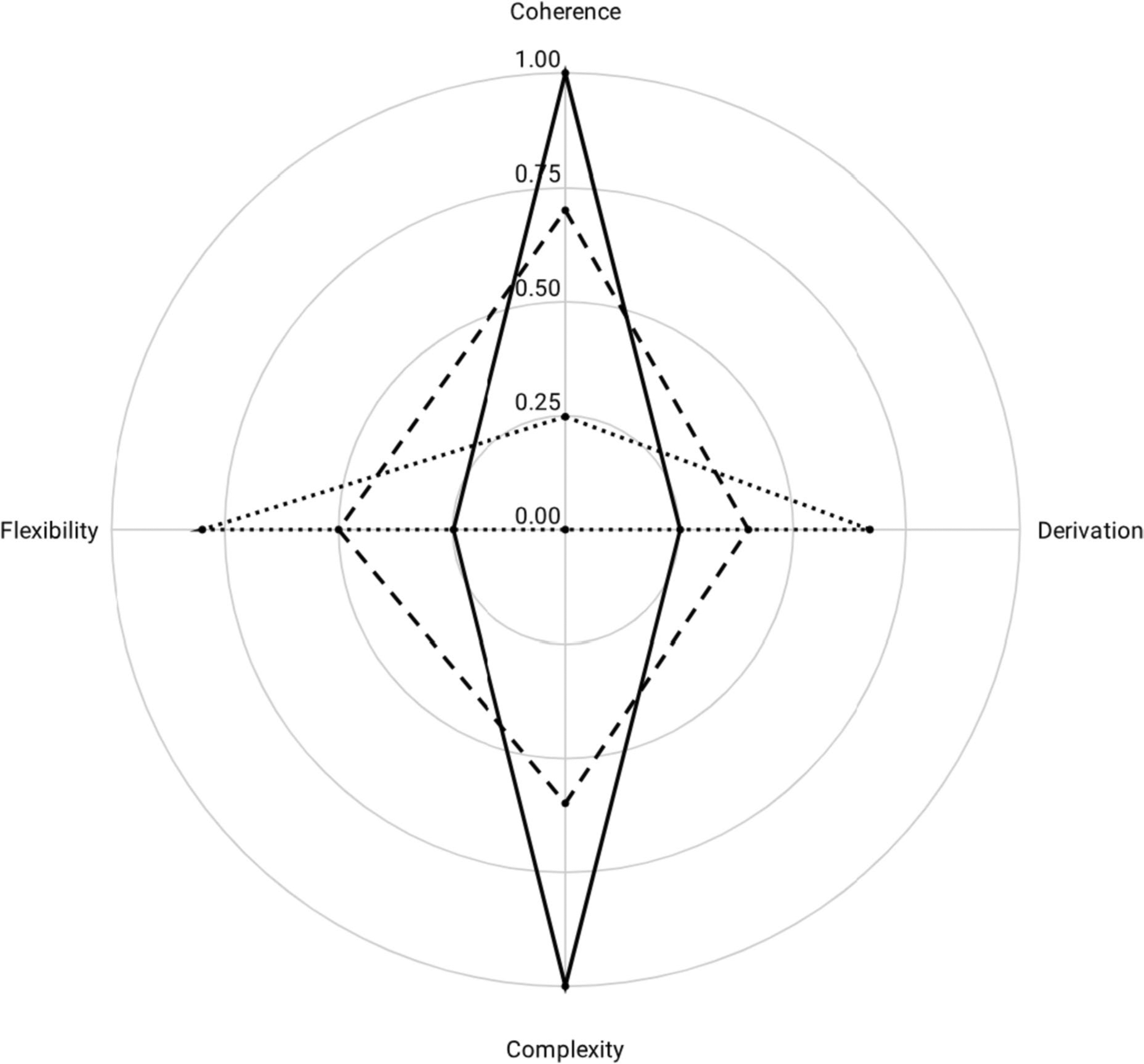
A Radar Chart Modeling Relatively High Levels of Coherence and Complexity, along with Relatively Low Levels of Flexibility and Derivation. *Note*: Initial assessment, dotted line; Reassessment – dashed line; Terminal model – solid line

Parallel to our use of radar charts to show multidimensional relationships between behavior and environment, RFT has more recently been described as a field of interactants, rather than individual frames (Barnes-Holmes et al., 2020). Heretofore, our analyses have emphasized the development of a larger profile across verbal behavior, frames of coordination, and deictic relations. However, multi-axial radar charts may also be used to fit a particular model (or shape). For example, Harte and Barnes-Holmes (2021) explain that relating relational networks requires a foundation of basic and midlevel relational frames (i.e., coordination, distinction, containment, temporality, and deictic) that are high in coherence and complexity, whereas low in derivation and flexibility. Figure 8 provides representative data of the HDML developing over time, along with a model of the ideal proportion of dynamic interaction between these four dimensions to serve as a framework for clinical guidance.

The frequency of discriminated responses are plotted on their respective axes of the HDML framework: coherence, complexity, derivation, and flexibility. The initial assessment (dotted line) shows *A* = 0.18, *R* = .09 (−.04, .08), for *Q* = 0.17. A reassessment (dashed line) shows *A* = 0.59, *R* = .05 (−.03, .03), for *Q* = 0.56. The difference between the two can be quantified as *Q* = 0.39 (see Table 4).

Unlike the models discussed above, the target for the HDML profile is not the maximum *Q* value. The target HDML profile (solid line) has *A* = 0.50, *R* = 0, and *Q* = 0.50. Note that the Q value at the time of reassessment (*Q* = 0.56) is larger than that of the targeted profile (*Q* = 0.50). As a result, visual analysis may be particularly useful for analyzing HDML.

The polygonal HDML profile at the time of the initial assessment shows increased flexibility and derivation, along with minimal coherence and no complexity. This might be the case for a speaker with autism whose language skills are severely restrictive (i.e., low coherence and complexity) or under a prepotent source of control (i.e., high flexibility and derivation). The reassessment shows a high degree of balance across the four domains, as flexibility and derivation have decreased somewhat, whereas coherence and complexity have expanded. Contrary to the previously discussed models, however, proportionality is not the priority for HDML.

The precise numeric value of the terminal HDML profile is less important than the shape of its polygonal profile, which—when plotted as in Fig. 8—depicts a rhombus with height (p) substantially greater than width (q). Other geographic models with varying levels of coherence, complexity, derivation, and flexibility may also be useful for conceptualizing human psychological events in terms of relating, orienting, and evoking within a given motivational context (Harte & Barnes-Holmes, 2021).

## Caveats of Radar Charts

Throughout this article, we have tried to demonstrate the utility of multi-axial radar charts for examining multidimensional relationships between environment and behavior. Using a methodology derived from the physical and biological sciences, we employed a multidimensional visualization strategy to compare the relative performance distributions of various environmental relations within a single visual graphic. The radar chart’s display of data as closed polygonal profiles affords the use of shape descriptors for quantitative analyses (Porter & Niksiar, 2018).

It should be noted that radar charts have several limitations to consider as we discuss their implications for studying complex behavior. Given our emphasis on area as a fundamental shape descriptor, we must acknowledge that the area of a polygonal profile is dependent on the ordering of the axes. For some analyses, the ordering of axes is more intuitive than others. Note that the radar chart in Fig. 3 was constructed from two continua, which led to a more natural ordering of axes. Order is also not a problem in Figs. 6 and 7, which only have three dimensions. However, for other analyses the ordering of axes is more arbitrary. For example, in Fig. 8 we purposefully placed *coherence* and *complexity* on opposite ends of the chart to create the more symmetric profile of a rhombus. Had we placed them adjacent to one another, the resulting trapezoidal profile would not only appear different, it would also yield a different area measure.

In addition, although multiple data series can be plotted on the same radar chart, too much data can quickly make a radar chart unreadable. For example, the hyperdimensional multilevel framework of RFT proposes five key levels of behavioral development (Barnes-Holmes et al., 2017; Barnes-Holmes et al., 2020; Harte & Barnes-Holmes, 2021). Attempting to plot all five levels of relational development across each of the four dimensions on the same radar chart is untenable. Instead, we suggest the creation of multiple charts to show each of the different levels. Likewise, although radar charts can be used to show change over time, we recommend limiting their use to two (e.g., pre- and post-) data series to avoid obfuscation.

## Extended Relations

Quantifying the interdependence of complex subject matters has been an important transition in our understanding of natural phenomena. The natural progression of our understanding and interpretation of data has evolved as our need to comprehend complex and interrelated phenomena became more pronounced. Consider the verbal operants—mands, tacts, intraverbals, and echoics—historically treated as exclusive units of analysis. Without accounting for covariation, they are interpreted in a rudimentary fashion that underestimates the interdependence of different but related sources of control. This continues to be the case when analyzing emergent verbal behavior on a two-dimensional chart. Whereas the supplementary sources of control are hidden on a line graph, they are revealed on a radar chart, which allows us to observe the growth of the interdependent verbal repertoire prior to the emergence of a new operant.

Within the natural environment, the convergence of controlling variables may be so ubiquitous that the purity of an operant becomes irrelevant (Michael et al., 2011). As the complexity of control increases, our measurement system must act accordingly. We have presented an alternative means of displaying complex data outside of those typically seen within the field of behavior analysis. Despite their limitations, multi-axial radar charts have clear implications for examining multiple relations. In conjunction with other research in this area, the multidimensional display of radar charts strengthens the relationship between modern behavior analysis and the other natural sciences (Barnes-Holmes et al., 2017; Belisle & Dixon, 2020; Bickerton, 2007; Hayes & Stanford, 2014).

Similar to two-dimensional line graphs, radar charts have the ability to show the dynamic relationship between independent and dependent variables. However, radar charts are unique in their ability to show the relationship between multiple independent and multiple dependent variables. As demonstrated above, specific polygonal profiles can serve as comparative models and help direct clinical intervention.

The polygonal profiles found on radar charts are also beneficial for quantitative analysis using shape descriptors. First moment of area accounts for both the density and distribution of stimulus relations. By providing a precise measure of complex controlling relations, first moment of area allows for intra-subject comparisons over time, can be used to evaluate the effects of intervention, and may otherwise serve as a basis for making data-based decisions.

**Fig. 9:**
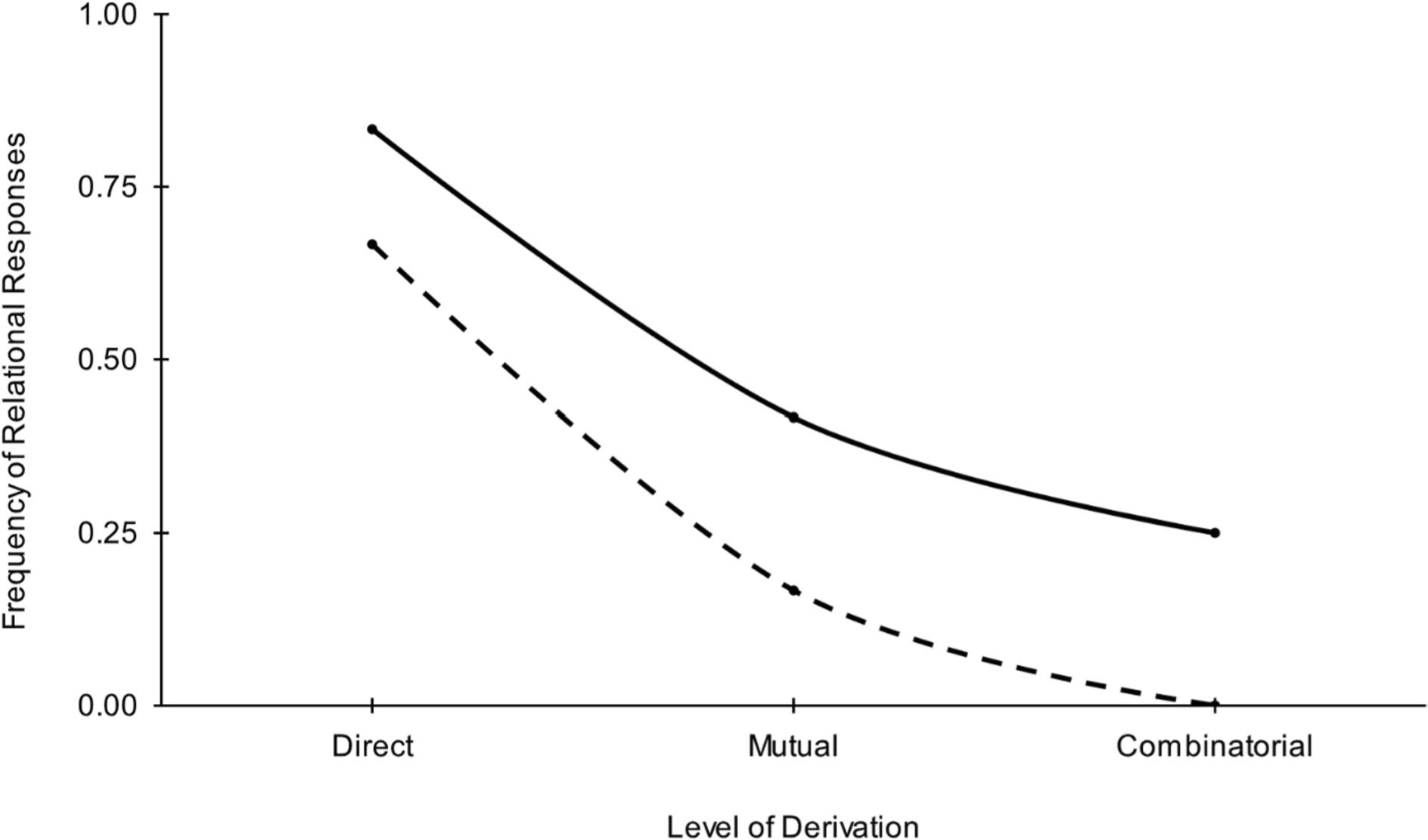
Two Derivation Gradients Showing the Results of Relational Operant Analyses of a Six-Year-Old Boy with Autism before (broken line) and after (solid line) One Year of Early Intensive Behavioral Intervention. *Note*: Initial assessment, dashed line; Reassessment – solid line

Future research should examine the potential clinical implications of such an analysis for conceptualizing mental disorders such as autism, in which stimulus overselectivity interferes with social communication among other adaptive skills. In addition, researchers should examine the correspondence between simple and complex environmental relations. For example, throughout the current article we argue for the use of first moment of area as a primary analytical unit. A sequential pattern of relating may occur throughout language acquisition, with directly taught relations precipitating mutual entailment, which later gives rise to combinatorial entailment due to its complexity and training history. Figure 9 displays the same data from Fig. 6 above plotted as a two-dimensional derivation gradient. Prior to intervention, the speaker’s relational responding shows a sharper loss of control across levels of derivation. Area under the curve (AUC), which was initially calculated as 0.25, increased to 0.48 after a year of EIBI. Future research should examine the relationship between first moment of area and the AUC that results from Fig. 9.

The current literature on language acquisition has made it abundantly clear that the standard approach to measuring behavior with two-dimensional line graphs is insufficient for analyzing complex human behavior. As the youngest of the natural sciences, behavior analysis can benefit from the example of its elders to address the growing pains of our field. Though unconventional, radar charts have offered potential applications within the realms of biological sciences and engineering. The utility of multi-axial radar charts to the field of behavior analysis ultimately lies in their explanatory power. Perhaps definitionally, complex behavior is that which necessitates a complex analysis.

## Figures and Tables

**Fig. 1:**
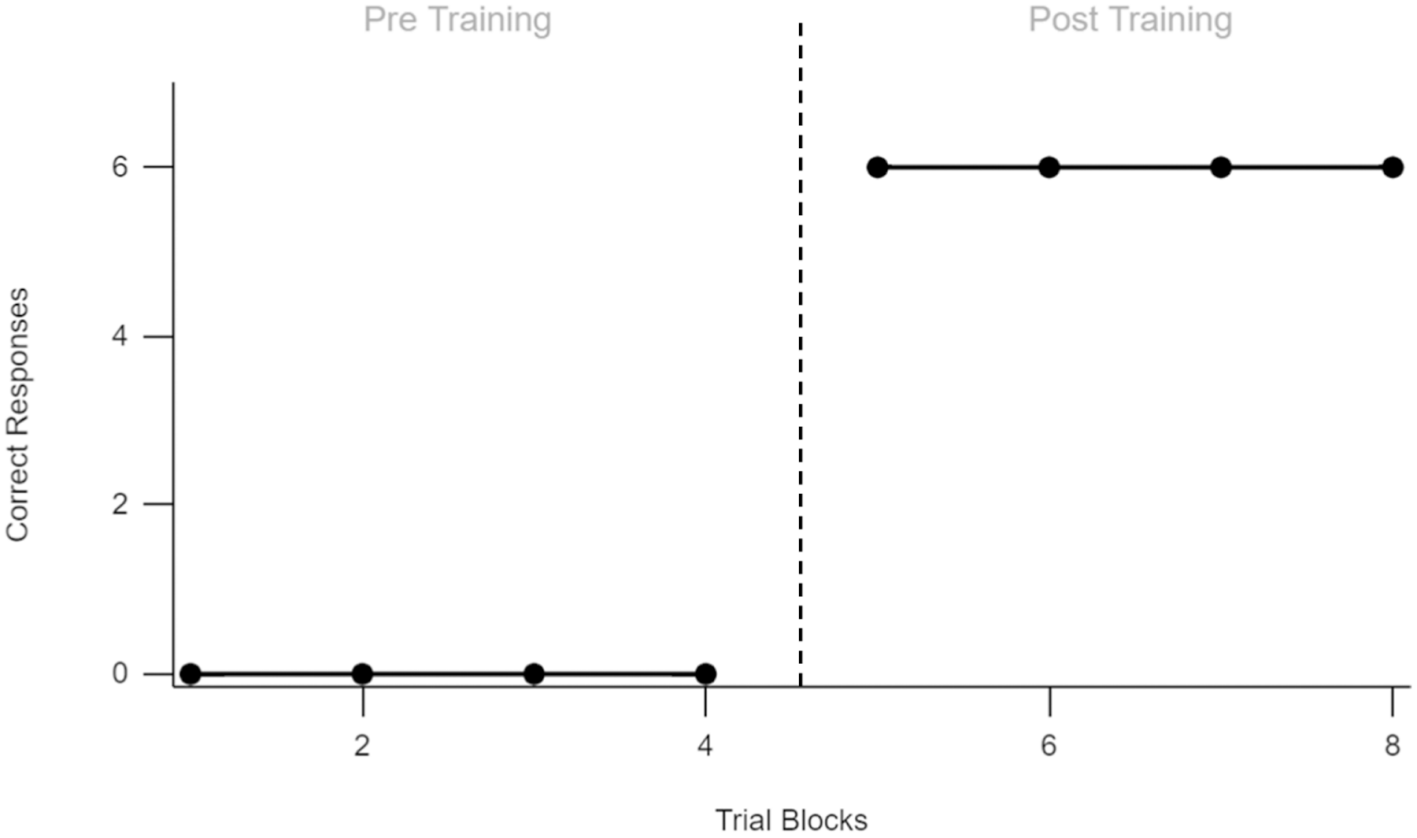
An Example of a Line Graph Redrawn from the Literature on the Emergence of Bidirectional Relations

**Table 1:** Measures of the Speaker’s Polygonal Language Profile over time

	Initial Assessment	Reassessment	Target Value
Area (*A*)	0.31	1.39	2.00
Centroidal Distance (*R*)	.30	.16	.00
First Moment of Area (*Q*)	0.22	1.16	2.00

**Table 2:** Measures of the Speaker’s Polygonal Relational Profile over time

	Initial Assessment	Reassessment	Target Value
Area (*A*)	0.05	0.29	1.30
Centroidal Distance (*R*)	.27	.17	.00
First Moment of Area (*Q*)	0.03	0.23	1.30

**Table 3:** Measures of the Speaker’s Polygonal Deictic Profile over time

	Initial Assessment	Reassessment	Target Value
Area (*A*)	0.02	0.27	1.30
Centroidal Distance (*R*)	.10	.12	.00
First Moment of Area (*Q*)	0.02	0.24	1.30

**Table 4:** Measures of the Speaker’s Polygonal HDML Profile over Time

	Initial Assessment	Reassessment	Target Value
Area (*A*)	0.18	0.59	0.50
Centroidal Distance (*R*)	.09	.05	.00
First Moment of Area (*Q*)	0.17	0.56	0.50

## Data Availability

All data generated or analyzed during this study are included in this published article.

## Footnotes

1 As Hayes (2002) explained, “The word ‘want’ comes from the Old Norse term *vant*, meaning literally ‘missing’” (p. 63).

2 For normalized data, *C* will always equal 1.

3 We employ a three-axis radar chart to explicate the differences across explicit and implicit relations, however the level of derivation may also be extended (see Harte et al., 2018, 2021). Relations that are more distal can be incorporated into such an analysis with the inclusion of additional axes.
